# Supplementary material for: A viral assembly inhibitor blocks SARS-CoV-2 replication in airway epithelial cells
Source: Commun Biol. 2024 Apr 22;7:486. doi: 10.1038/s42003-024-06130-8 (PMC11035691; doi:10.1038/s42003-024-06130-8)

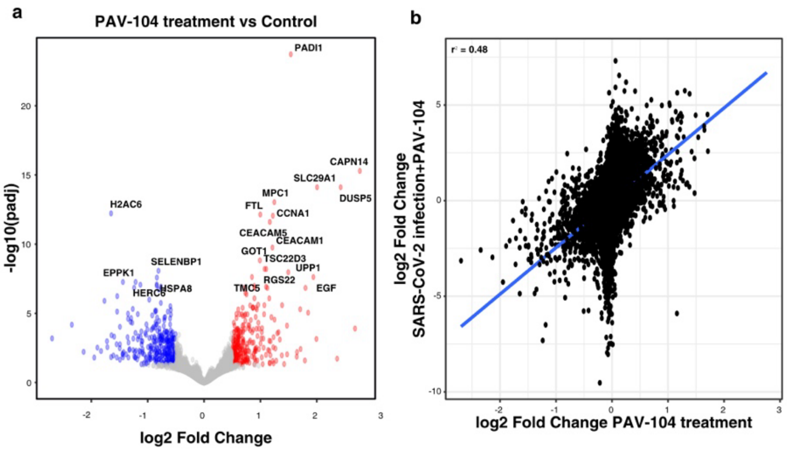

Supplementary Figure 1. The effect of PAV-104 treatment on the primary AEC transcriptome. (a) Volcano plot showing the proportion of DEGs in the setting of PAV-104 treatment. DEGs (FDR < 0.05) with Log2(fold change) > 0 are indicated in red. DEGs (FDR < 0.05) with Log2(fold change) < 0 are indicated in blue. Non-significant DEGs are indicated in grey. (b) Correlation between PAV-104 treatment and SARS-CoV-2 infection with PAV-104 treatment. Our analysis revealed significant down-regulation of 454 genes and up-regulation of 489 genes due to PAV-104 treatment alone. An examination of the transcriptomic relatedness of PAV-104 treatment alone to SARS-CoV-2 infection in the presence of PAV-104 revealed a moderate correlation between gene expression profiles (R^2 = 0.48). However, there are numerous genes that were differentially modulated,

suggesting that a substantial fraction of the transcriptomic perturbation results from an interaction between SARS-CoV-2 and PAV-104 (although inter-donor variability may also play a role).

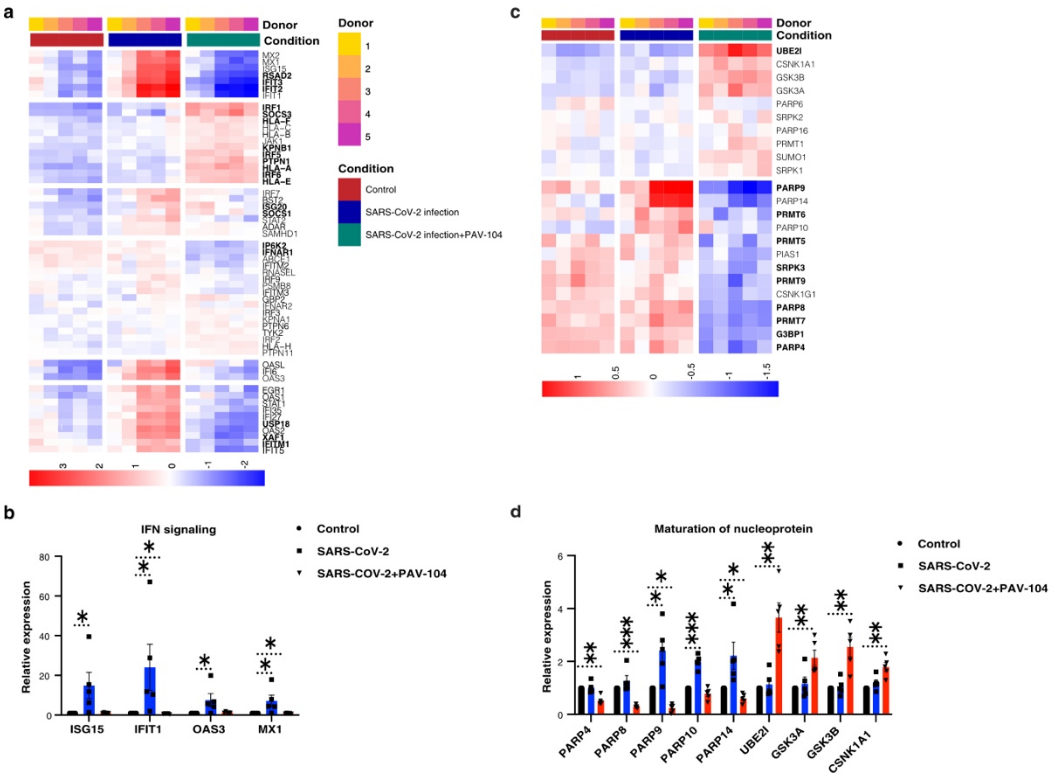

Supplementary Figure 2. Gene expression signatures of IFN signaling and maturation of nucleoprotein pathways in response to SARS-CoV-2 infection and PAV-104 treatment. (a) Heat map depicting the regulation of all genes included in the IFNalpha/beta signaling pathway. The bold indicates statistical significance with FDR < 0.05 and an absolute log2 fold change > 1. (b) The expression of select ISGs was verified by RT-qPCR. n=5 biologically independent donors. (c) Heat map depicting the regulation of all genes involved in the maturation of nucleoprotein pathway. The bold indicates statistical significance with FDR < 0.05 and an absolute log2 fold change > 1. (d) The expression of select genes associated with the maturation of nucleoprotein signaling pathway was verified by RT-qPCR. n=5 biologically independent donors. Data are

representative of the results as mean ± SEM. Statistical significance was determined by paired *t* test. $p \leq 0.05$ [*], $p \leq 0.01$ [**], $p \leq 0.001$ [***], $p \leq 0.0001$ [****].

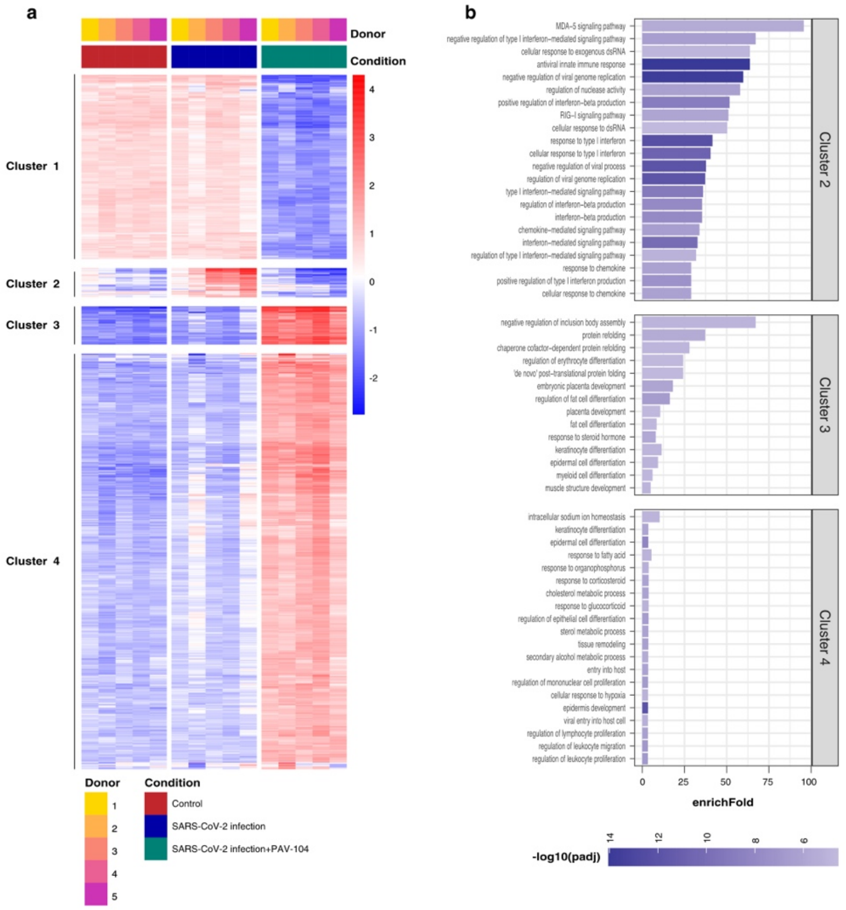

Supplementary Figure 3. Clustering analysis of the 1000 most highly-variable genes across samples. (a) Heat map depicting normalized expression levels of the 1000 most variably-expressed genes across samples, clustered into 4 groups based on unsupervised analysis of differential expression between treatment conditions. (b) Gene Ontologies (GO) analysis for the cluster modules.

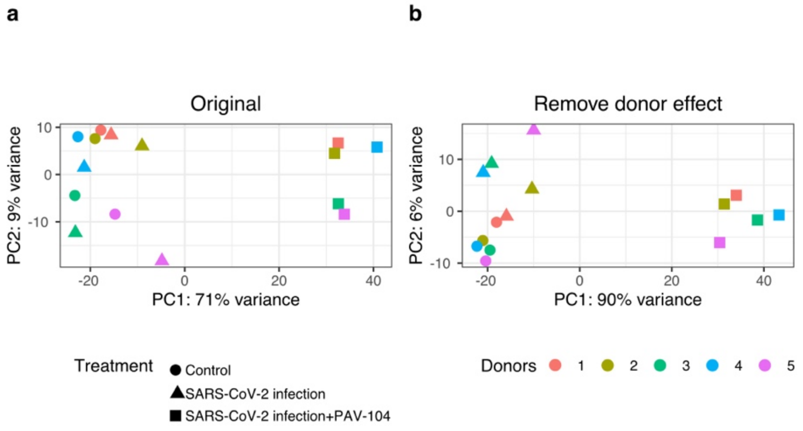

Supplementary Figure 4. Principle component analysis (PCA) visualizing donor effects in RNA-seq data.

Supplementary Figure 5. Source data for Figure 6a Pellet membrane

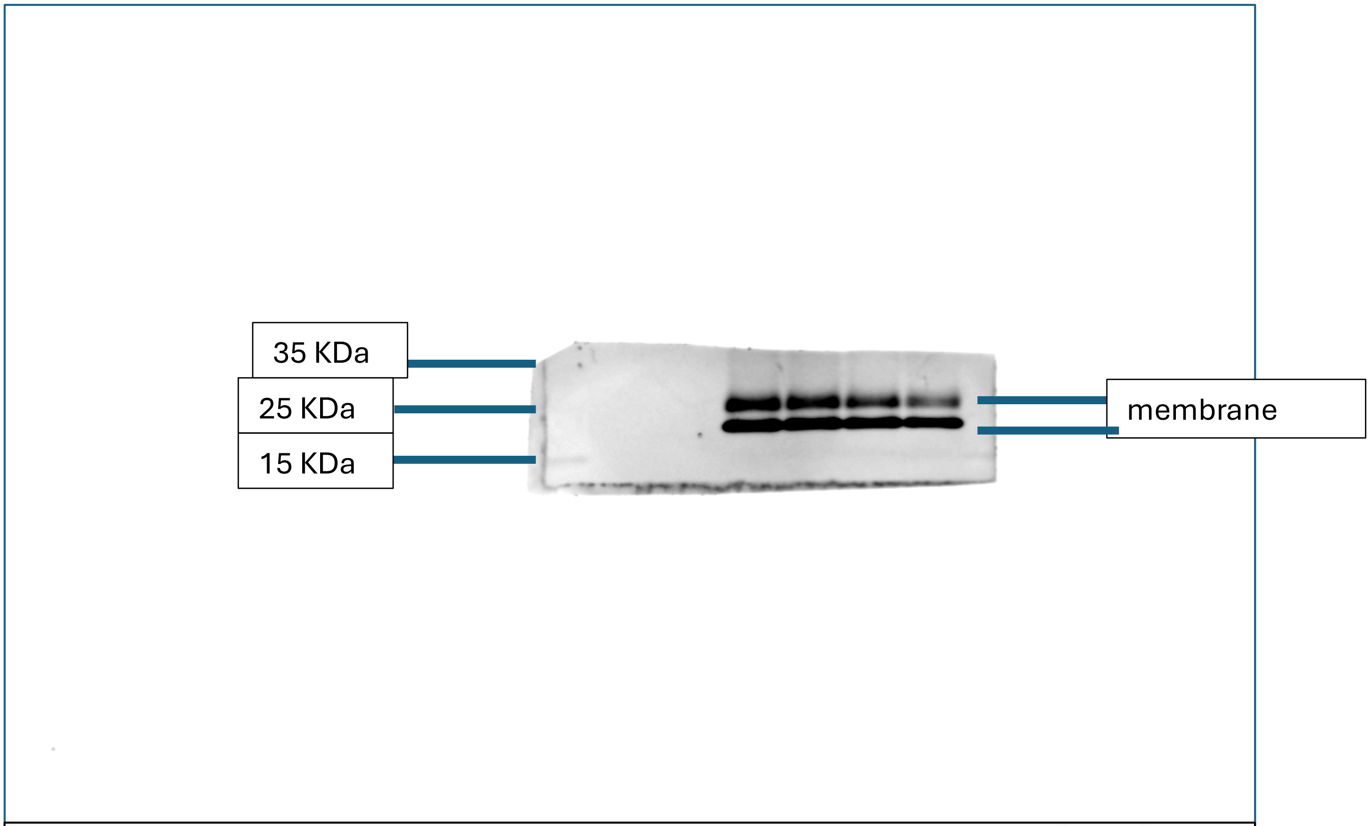

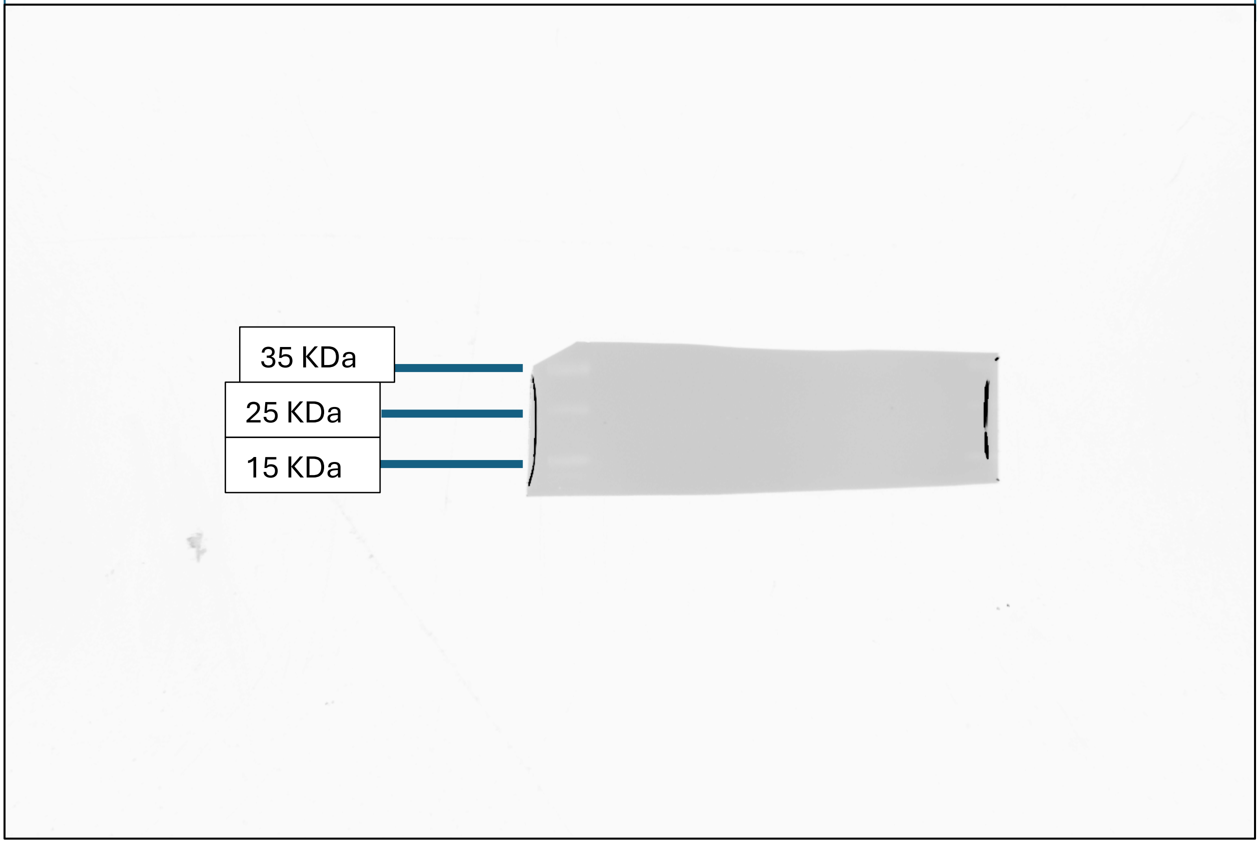

Supplementary Figure 6. Source data for Figure 6a Pellet nucleocapsid

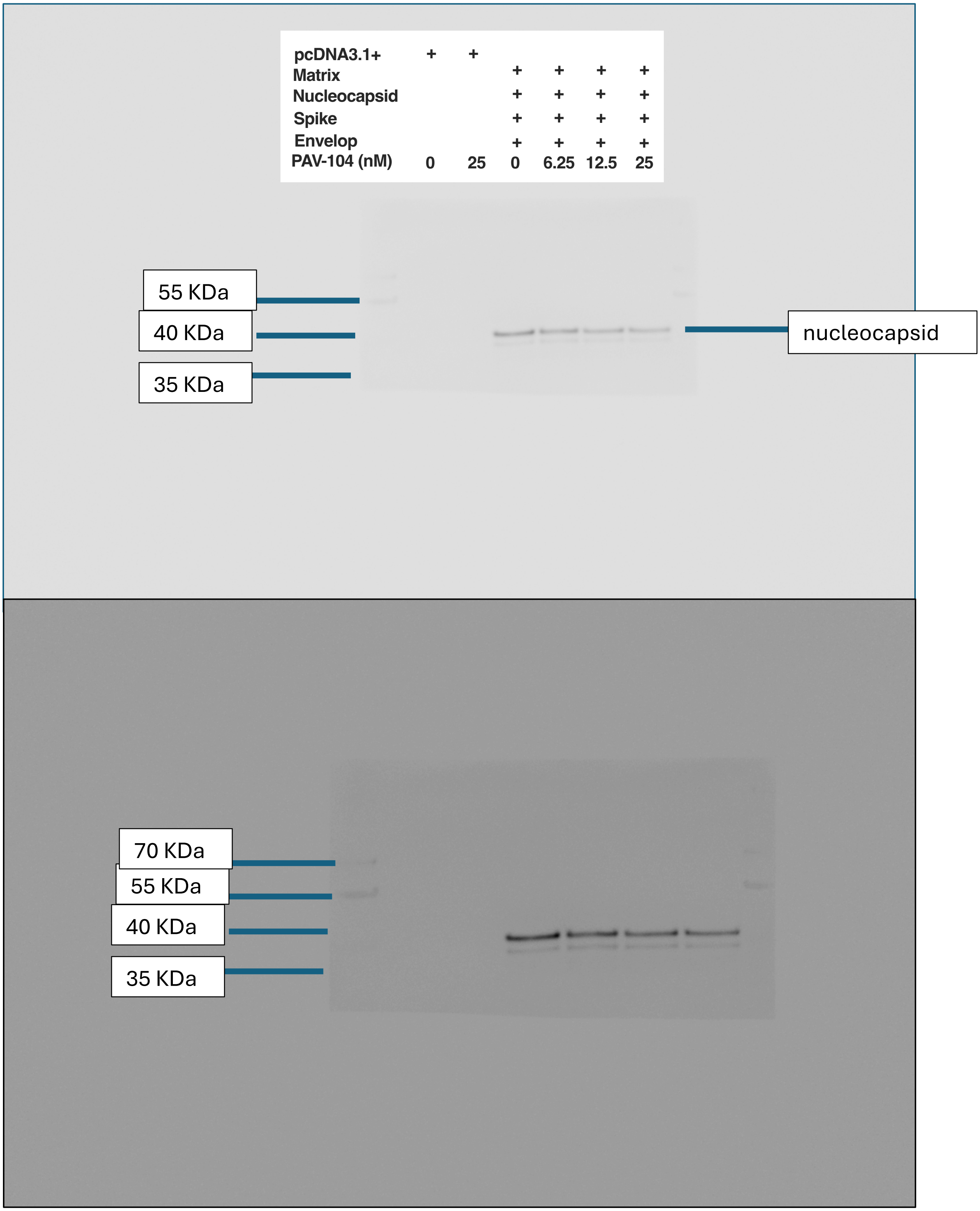

Supplementary Figure 7. Source data for Figure 6a Pellet spike

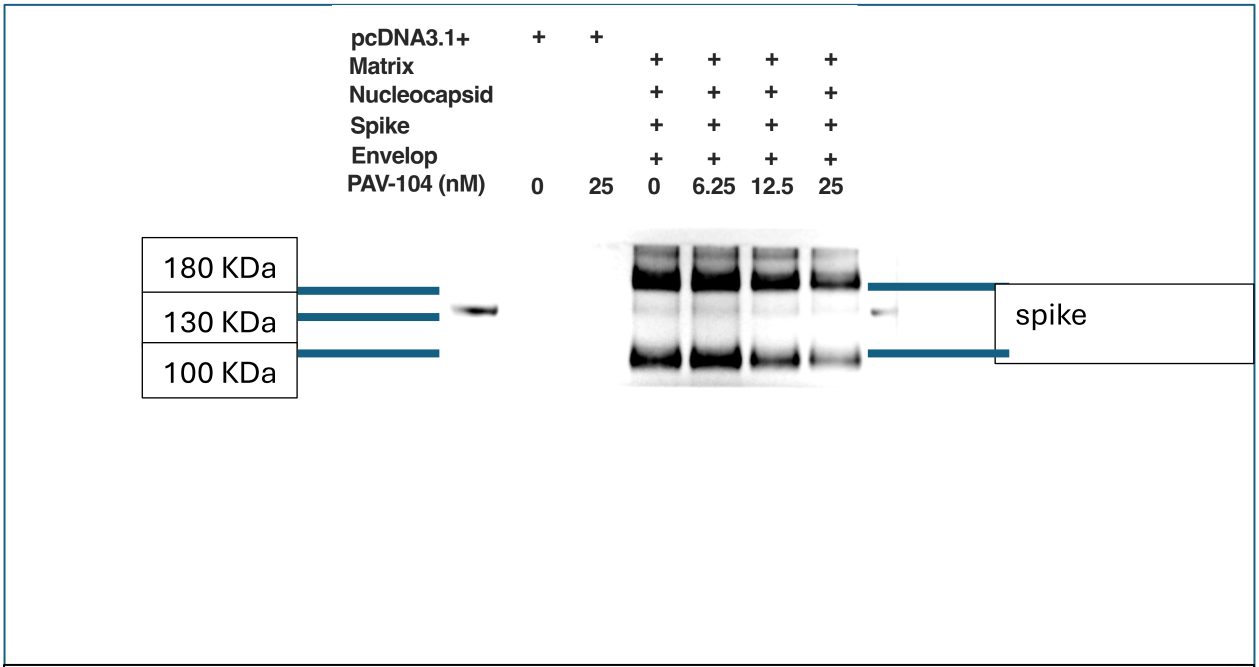

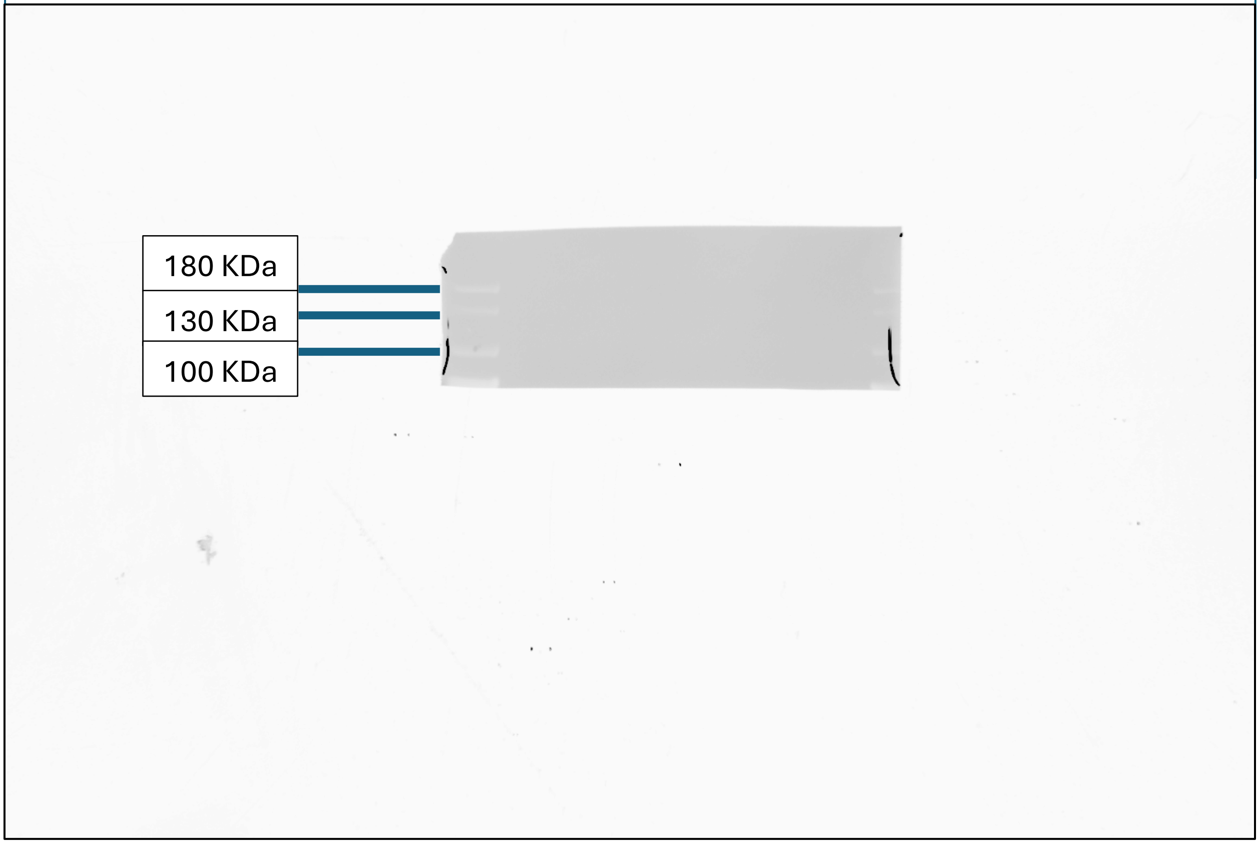

Supplementary Figure 8. Source data for Figure 6a cell lysates membrane

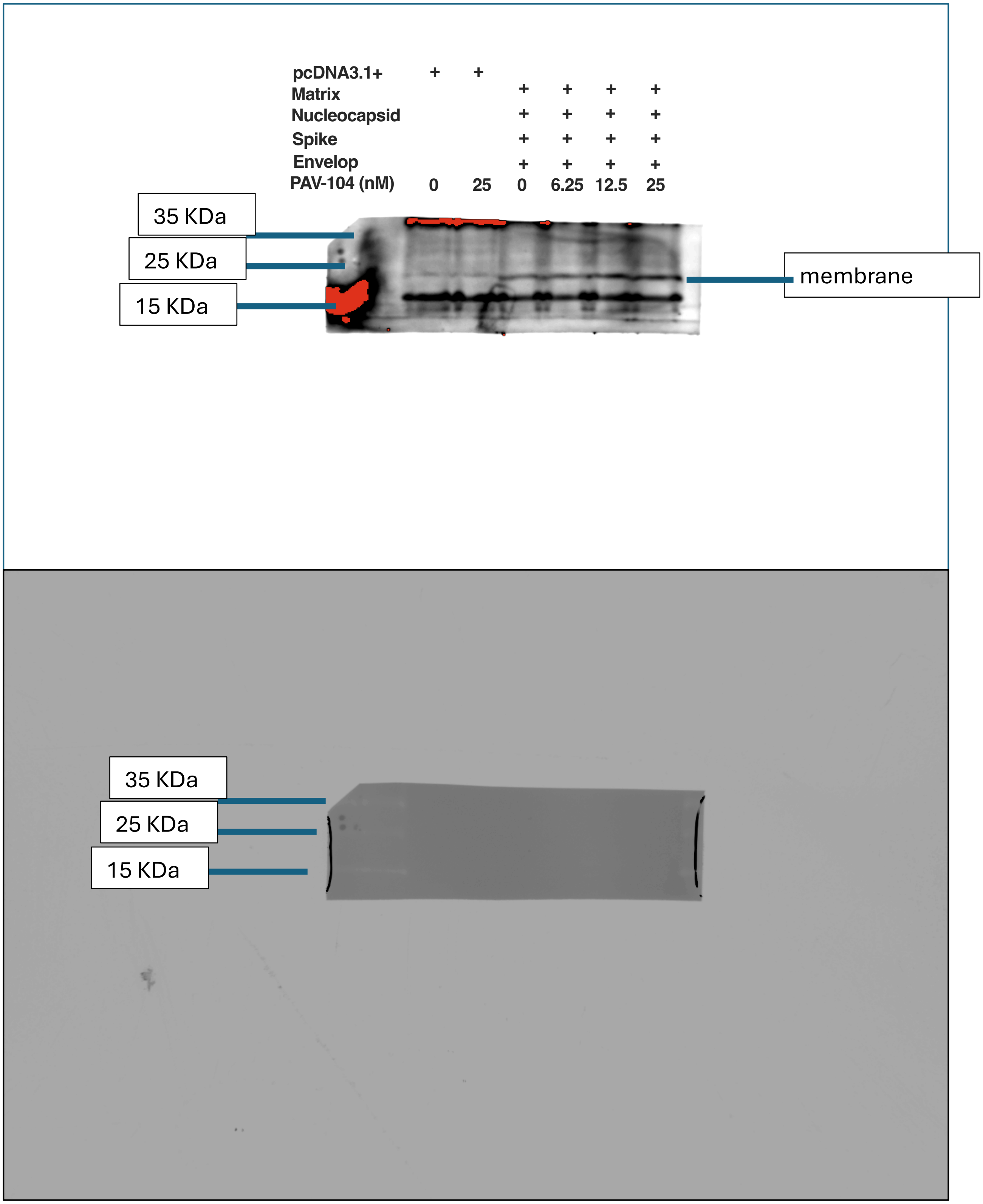

Supplementary Figure 9. Source data for Figure 6a cell lysates nucleocapsid

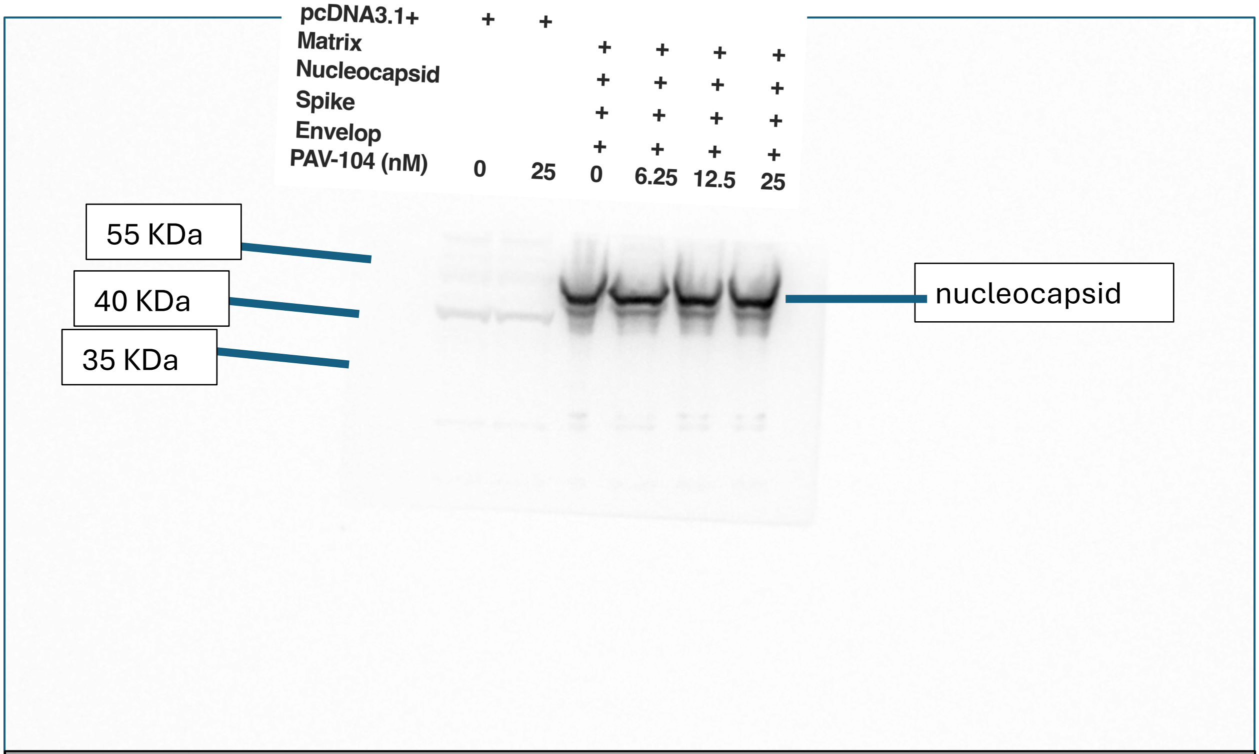

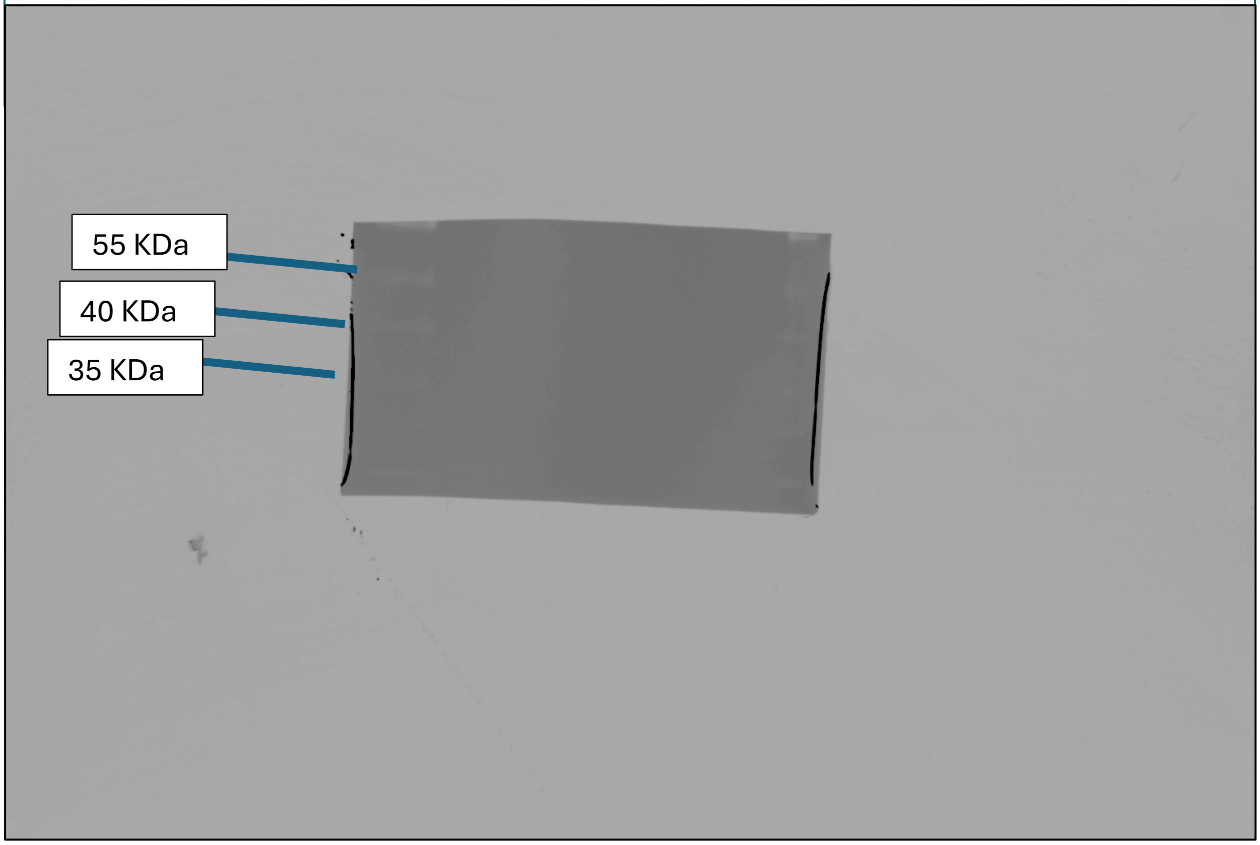

Supplementary Figure 10. Source data for Figure 6a cell lysates spike

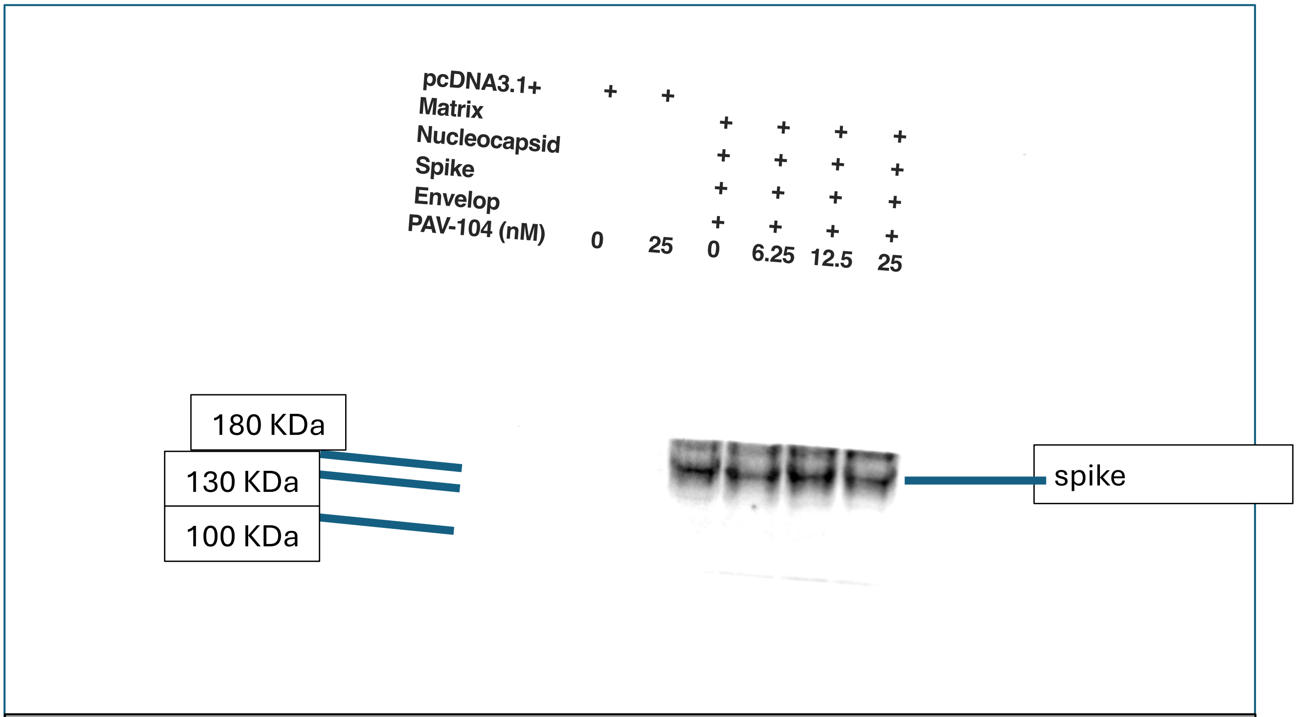

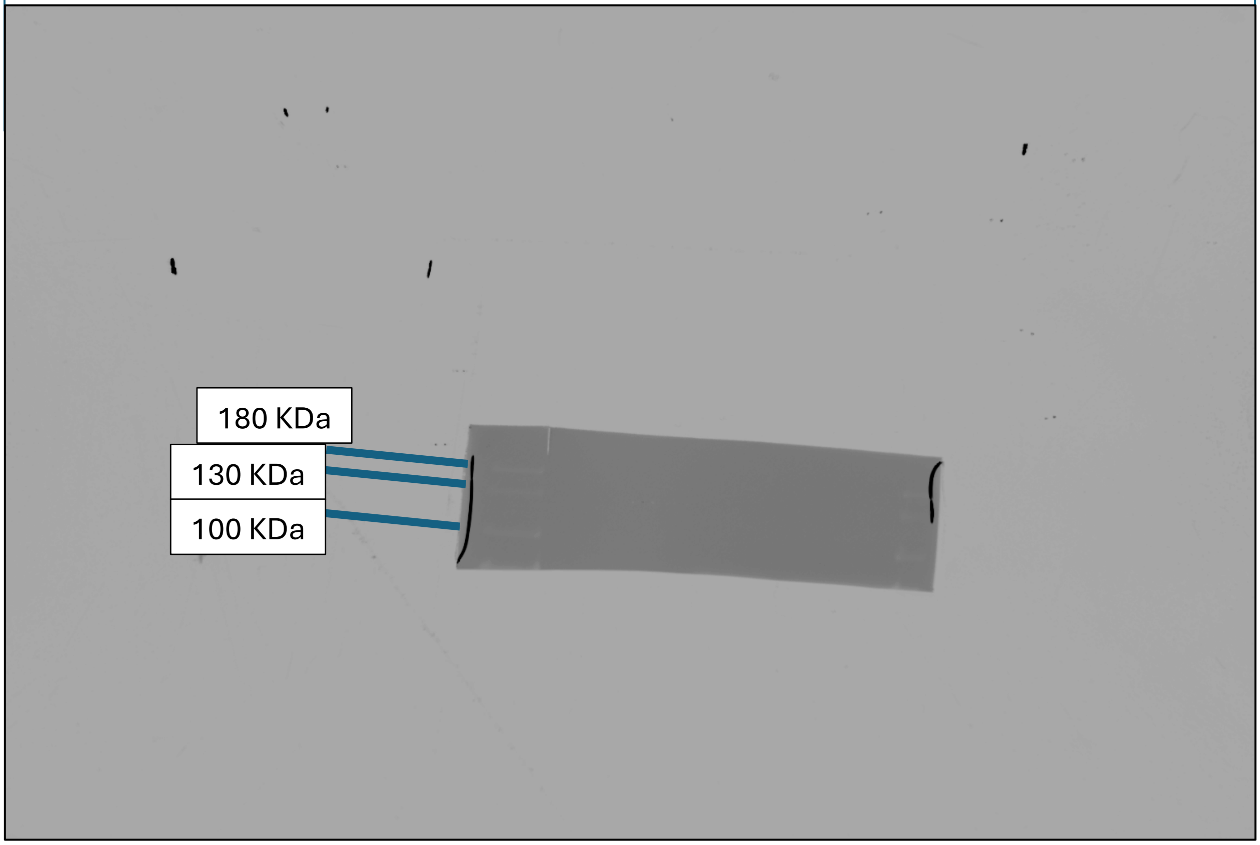

Supplementary Figure 11. Source data for Figure 6a cell lysates beta-actin

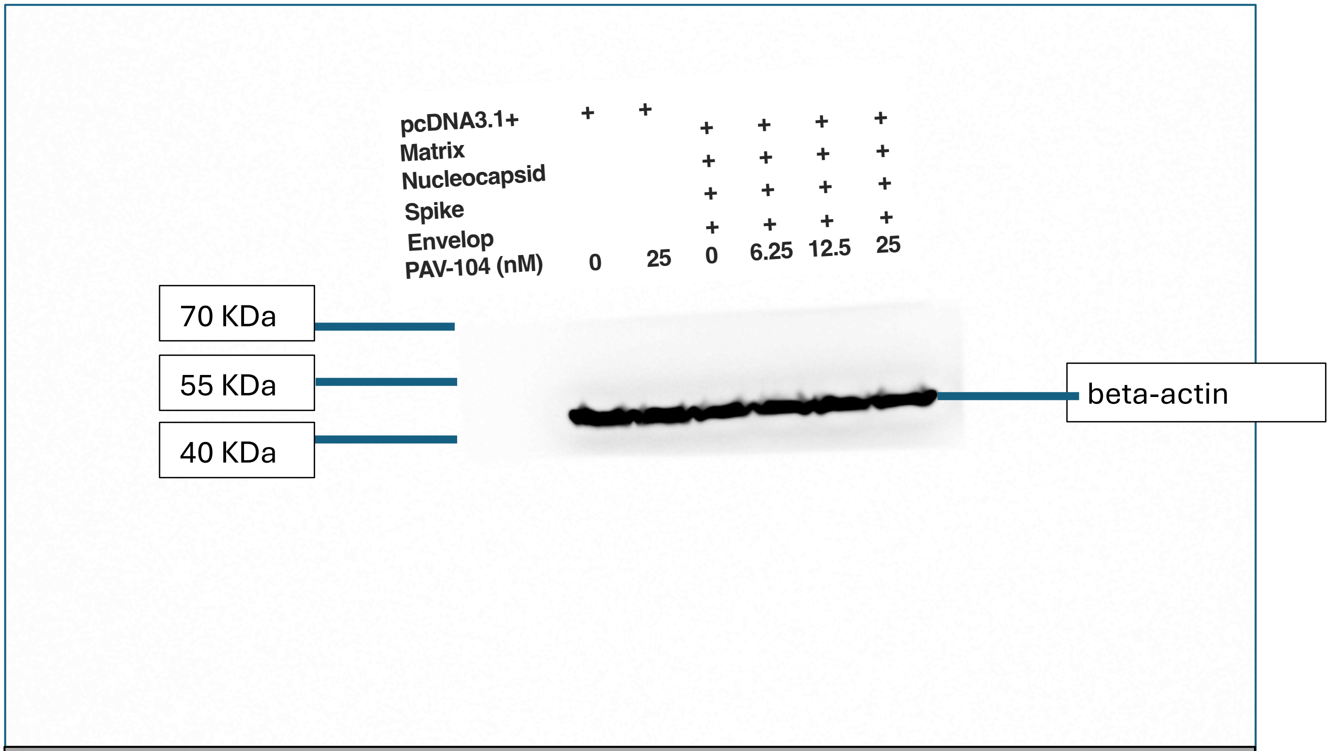

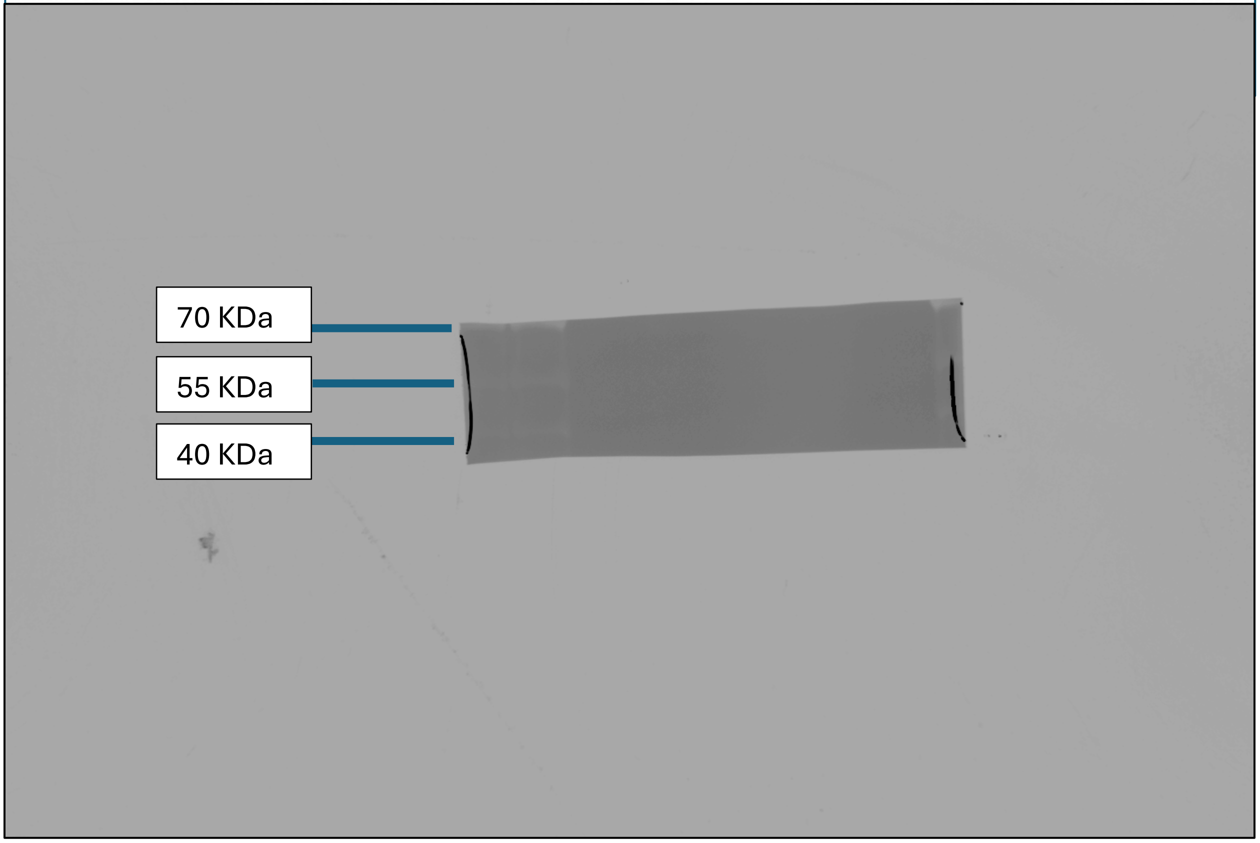

Supplementary Figure 12. Source data for Figure 7b nucleocapsid

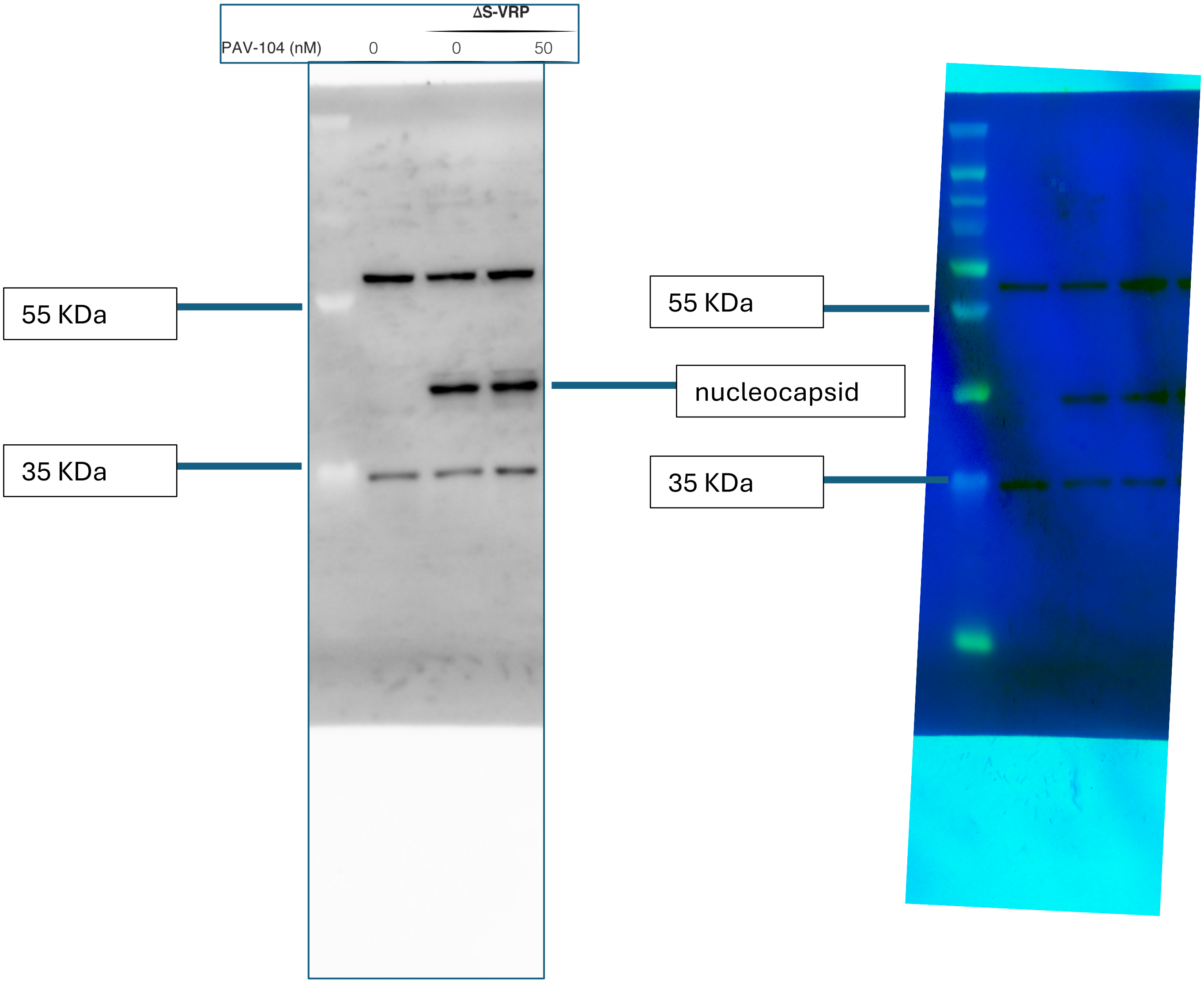

Supplementary Figure 13. Source data for Figure 7b GAPDH

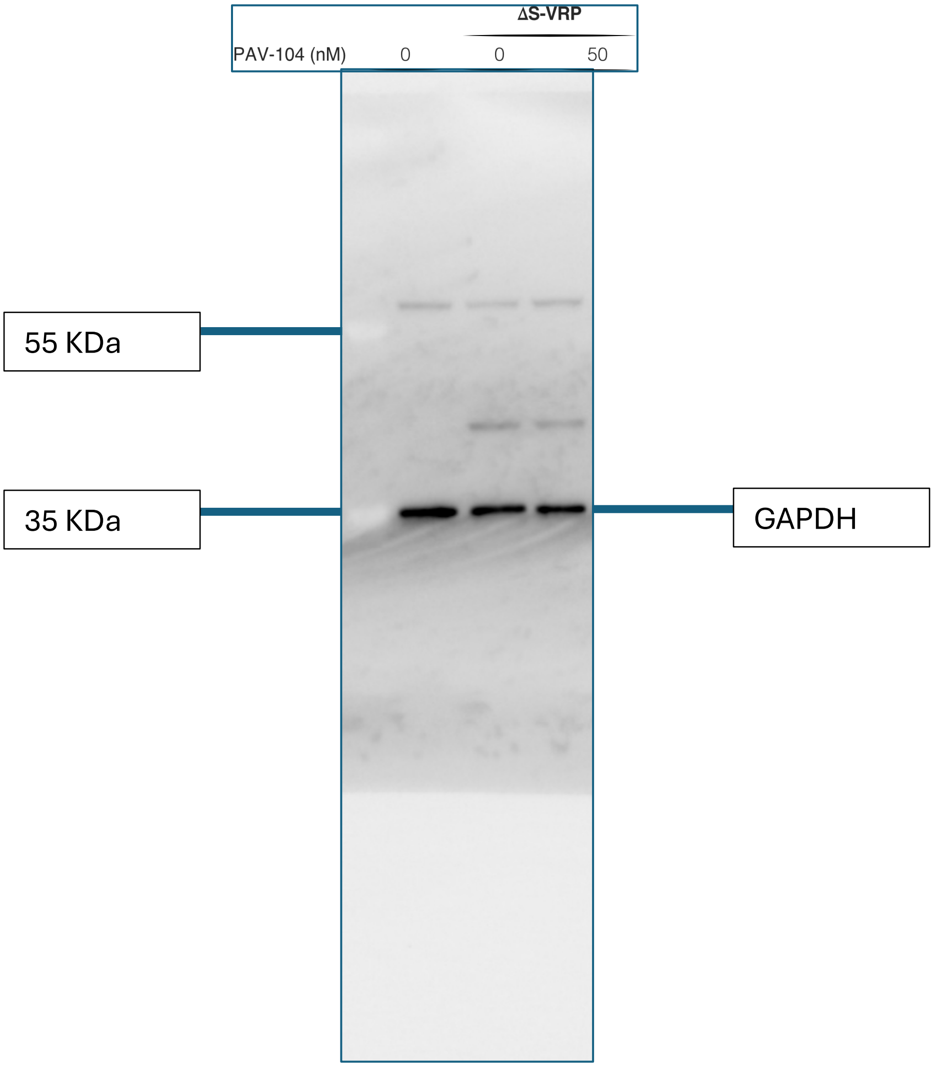

Supplement: Supplementary file 1 — Supplementary Information [file 42003_2024_6130_MOESM1_ESM.pdf]
