# Supplementary material for: A viral assembly inhibitor blocks SARS-CoV-2 replication in airway epithelial cells
Source: Commun Biol. 2024 Apr 22;7:486. doi: 10.1038/s42003-024-06130-8 (PMC11035691; doi:10.1038/s42003-024-06130-8)
Supplement: Supplementary file 2 — Description of Additional Supplementary Files [file 42003_2024_6130_MOESM2_ESM.pdf]

## Description of Additional Supplementary Files

**File name:** Supplementary Data 1
**Description:** The differentially-expressed gene lists by SARS-CoV-2 infection alone.

**File name:** Supplementary Data 2
**Description:** The differentially-expressed gene lists by SARS-CoV-2 infection in the presence of PAV-104.

**File name:** Supplementary Data 3
**Description:** The differentially-expressed gene lists by SARS-CoV-2 infection in the presence of PAV-104, when compared with SARS-CoV-2 infection alone.

**File name:** Supplementary Data 4
**Description:** The GSEA pathway enrichment data.

**File name:** Supplementary Data 5
**Description:** The source data behind the graphs in the paper.
