# Supplementary material for: A viral assembly inhibitor blocks SARS-CoV-2 replication in airway epithelial cells
Source: Commun Biol. 2024 Apr 22;7:486. doi: 10.1038/s42003-024-06130-8 (PMC11035691; doi:10.1038/s42003-024-06130-8)
Supplement: Supplementary file 8 — Reporting Summary [file 42003_2024_6130_MOESM8_ESM.pdf]

nature portfolio

Corresponding author(s): Satish K. Pillai

Last updated by author(s): Mar 18, 2024

Reporting Summary

Nature Portfolio wishes to improve the reproducibility of the work that we publish. This form provides structure for consistency and transparency in reporting. For further information on Nature Portfolio policies, see our Editorial Policies and the Editorial Policy Checklist.

Statistics

For all statistical analyses, confirm that the following items are present in the figure legend, table legend, main text, or Methods section.

n/a	Confirmed	
☐	☒	The exact sample size (*n*) for each experimental group/condition, given as a discrete number and unit of measurement
☐	☒	A statement on whether measurements were taken from distinct samples or whether the same sample was measured repeatedly
☐	☒	The statistical test(s) used AND whether they are one- or two-sided<br>*Only common tests should be described solely by name; describe more complex techniques in the Methods section.*
☒	☐	A description of all covariates tested
☒	☐	A description of any assumptions or corrections, such as tests of normality and adjustment for multiple comparisons
☐	☒	A full description of the statistical parameters including central tendency (e.g. means) or other basic estimates (e.g. regression coefficient) AND variation (e.g. standard deviation) or associated estimates of uncertainty (e.g. confidence intervals)
☐	☒	For null hypothesis testing, the test statistic (e.g. *F*, *t*, *r*) with confidence intervals, effect sizes, degrees of freedom and *P* value noted<br>*Give P values as exact values whenever suitable.*
☒	☐	For Bayesian analysis, information on the choice of priors and Markov chain Monte Carlo settings
☒	☐	For hierarchical and complex designs, identification of the appropriate level for tests and full reporting of outcomes
☒	☐	Estimates of effect sizes (e.g. Cohen's *d*, Pearson's *r*), indicating how they were calculated

Our web collection on statistics for biologists contains articles on many of the points above.

Software and code

Policy information about availability of computer code

Data collection	RNA concentration and quality was measured using High Sensitivity RNA ScreenTape Analysis (Agilent, 5067-1500). cDNA libraries were constructed and sequencing was performed by Novogene using their mRNA sequencing protocol.
Data analysis	The raw RNA sequencing data were aligned to the human genome (GRCh38) using STAR (version 2.7.3a). Analysis of differential expression was performed using DESeq2 according to a standard protocol. Genes with adjusted P-value < 0.05 were considered as significantly differentially expressed. Gene set enrichment analysis was performed using the fgsea package (version 1.22.0) in R. The Reactome database (version 7.5.1) was downloaded from MSigDB (https://www.gsea-msigdb.org). Reads were also aligned to the SARS-CoV-2 isolate WA-1 and analyzed using the QIAGEN CLC Genomics Workbench. To measure the frequency of infected cells, randomly-selected areas were imaged. Each treatment had three replicates. The FITC-positive cells and DAPI-positive cells were quantified using CellProfiler software. The same threshold value was applied to the images of each area. Quantification of the western blots was carried out with Image J software. Statistical analysis was performed using GraphPad Prism version 8 software. Source codes and accompanying information can be found at https://github.com/ldu1/PAV104.git

For manuscripts utilizing custom algorithms or software that are central to the research but not yet described in published literature, software must be made available to editors and reviewers. We strongly encourage code deposition in a community repository (e.g. GitHub). See the Nature Portfolio guidelines for submitting code & software for further information.

Data

Policy information about availability of data

All manuscripts must include a data availability statement. This statement should provide the following information, where applicable:

- Accession codes, unique identifiers, or web links for publicly available datasets
- A description of any restrictions on data availability
- For clinical datasets or third party data, please ensure that the statement adheres to our policy

The differentially-expressed gene lists are showed in Supplementary Data 1-3. The GSEA pathway enrichment data are showed in Supplementary Data 4. Sequencing data are available in the NCBI Gene Expression Ominibus under the GEO accession number GSE261002. All of the data generated or analyzed during this study are included in this published article or are available from the corresponding author upon reasonable request. The source data underlying the graphs in the figure are shown in Supplementary Data 5. Uncropped western blots are in Supplementary Figures.

Research involving human participants, their data, or biological material

Policy information about studies with human participants or human data. See also policy information about sex, gender (identity/presentation), and sexual orientation and race, ethnicity and racism.

Reporting on sex and gender	This information has not been collected.
Reporting on race, ethnicity, or other socially relevant groupings	This information has not been collected.
Population characteristics	See above.
Recruitment	Human unused donor tracheobronchial tissue was obtained at the time of lung transplant.
Ethics oversight	The studies involving human participants were reviewed and approved by the Human Research Protection Program, University of California, San Francisco. The patients/participants provided their written informed consent to participate in this study.

Note that full information on the approval of the study protocol must also be provided in the manuscript.

Field-specific reporting

Please select the one below that is the best fit for your research. If you are not sure, read the appropriate sections before making your selection.

☒ Life sciences ☐ Behavioural & social sciences ☐ Ecological, evolutionary & environmental sciences

For a reference copy of the document with all sections, see nature.com/documents/nr-reporting-summary-flat.pdf

Life sciences study design

All studies must disclose on these points even when the disclosure is negative.

Sample size	No sample size calculation was performed. The data presented in this research were derived from three biological replicates, with the exception of the sample used for Figure 7A's control column.
Data exclusions	No data were excluded.
Replication	Replicate experiments were successful
Randomization	Participants were chosen based on explanted healthy lungs.
Blinding	No blinding was performed in this study.

Reporting for specific materials, systems and methods

We require information from authors about some types of materials, experimental systems and methods used in many studies. Here, indicate whether each material, system or method listed is relevant to your study. If you are not sure if a list item applies to your research, read the appropriate section before selecting a response.

Materials & experimental systems

n/a	Involved in the study	
☐	☒	Antibodies
☐	☒	Eukaryotic cell lines
☒	☐	Palaeontology and archaeology
☐	☒	Animals and other organisms
☒	☐	Clinical data
☒	☐	Dual use research of concern
☒	☐	Plants

Methods

n/a	Involved in the study	
☒	☐	ChIP-seq
☒	☐	Flow cytometry
☒	☐	MRI-based neuroimaging

Antibodies

Antibodies used

1. Rabbit polyclonal anti-SARS-CoV-2 Nucleocapsid antibody, GeneTex, Cat# GTX135357, RRID A68464B_28, 1:1000 dilution;
2. Goat polyclonal anti-Rabbit IgG (H+L) Secondary antibody, FTIC, ThermoFisher Scientific, Cat# 65-6111, RRID AB_2533966, 1:100 dilution;
3. Rabbit polyclonal anti-SARS-CoV-2 Matrix antibody, ThermoFisher Scientific, Cat# PA1-41160, RRID AB_1087198, 1:1000 dilution;
4. Rabbit polyclonal anti-SARS-CoV-2 Nucleocapsid antibody, Rockland Immunochemicals, Cat# 200-401-A50, 1:1000 dilution;
5. Rabbit polyclonal anti-SARS-CoV-2 Envelope antibody, ThermoFisher Scientific, Cat# PA5-112047, RRID AB_2866783, 1:1000 dilution;
6. Rabbit polyclonal anti-SARS-CoV-2 Spike protein S1/S2 antibody, ThermoFisher Scientific, Cat# PA5-112048, RRID AB_2866784, 1:1000 dilution;
7. Mouse monoclonal anti-SARS-CoV-2 Spike antibody, GeneTex, Cat# GTX632604, RRID AB_2864418, 1:1000 dilution, clone 1A9;
8. Anti-Mouse IgG HRP conjugated secondary antibody, Cell Signaling Technology, Cat# 7076S, 1:5000 dilution;
9. Anti-Rabbit IgG HRP conjugated secondary antibody, Cell Signaling Technology, Cat# 7074S, 1:5000 dilution;
10. Rabbit monoclonal β-Actin HRP conjugated antibody, Cell Signaling Technology, Cat# 12620S, 1:10000 dilution;

Validation

All antibodies are commercially available and were commercially validated.

Eukaryotic cell lines

Policy information about cell lines and Sex and Gender in Research

Cell line source(s)

Calu-3 cells (ATCC-HTB-55); Vero E6 cells (ATCC-CRL-1586); HEK29T cells (ATCC-CRL-3216);

Authentication

None of the cell lines used were authenticated.

Mycoplasma contamination

All cell lines tested negative for mycoplasma contamination.

Commonly misidentified lines
(See ICLAC register)

Not applicable.

Animals and other research organisms

Policy information about studies involving animals; ARRIVE guidelines recommended for reporting animal research, and Sex and Gender in Research

Laboratory animals

The study did not involve laboratory animals.

Wild animals

The study did not involve wild animals.

Reporting on sex

This information has not been collected.

Field-collected samples

Primary AECs were obtained and cultured as previously described28. Briefly, Human unused donor tracheobronchial tissue was obtained at the time of lung transplant. The tissue was washed and placed in DMEM with 0.1% protease and antibiotics overnight at 4ºC. The next day, the solution was agitated, and the remaining tissue was removed. Cell pellets were treated with 0.05% trypsin-EDTA, then filtered through a cell strainer. Cells were plated onto 6mm/0.4mm Transwell ALI insert after treatment with FNC coating mixture. 10% FBS in DMEM and ALI media were added in equal volumes to each basal compartment and cultures were incubated at 37ºC with 5% CO_2. The next day, the media was removed and both compartments were washed with PBS and antibiotics. ALI media was then added to each basal compartment and changed every three days for at least 28 days until differentiated airways were ready for use .

Ethics oversight

The studies involving human participants were reviewed and approved by the Human Research Protection Program, University of California, San Francisco. The patients/participants provided their written informed consent to participate in this study.

Note that full information on the approval of the study protocol must also be provided in the manuscript.
